# Positive predictive value of ERBB2 copy number gain by tissue or circulating tumor DNA next-generation sequencing across advanced cancers

**DOI:** 10.18632/oncotarget.28188

**Published:** 2022-02-02

**Authors:** Ami N. Shah, Ashwin Sunderraj, Brian Finkelman, Sharlene H. See, Andrew A. Davis, Lorenzo Gerratana, Firas Wehbe, Neelima Katam, Deva Mahalingam, William J. Gradishar, Amir Behdad, Luis Blanco, Massimo Cristofanilli

**Affiliations:** ^1^Robert H. Lurie Comprehensive Cancer Center of Northwestern University, Chicago, IL, USA; ^2^Department of Pathology, The Johns Hopkins Medical Institutions, Baltimore, MD, USA; ^3^Siteman Cancer Center of Washington University, St. Louis, MO, USA; ^4^Department of Medical Oncology, Centro di Riferimento Oncologico (CRO), IRCCS, Aviano, Italy

**Keywords:** ERBB2, HER2, next-generation sequencing, copy number gain, circulating tumor DNA

## Abstract

Background: The correlation of ERBB2 copy number gain (CNG) from tissue or circulating tumor DNA (ctDNA) by next-generation sequencing (NGS) with standard HER2 tissue evaluation is not well understood.

Materials and Methods: We retrospectively identified patients with ERBB2 CNG on commercial NGS. We described their clinical-pathologic features and calculated the positive predictive value (PPV) of ERBB2 CNG by NGS for HER2-positivity by IHC and FISH testing.

Results: 176 patients had NGS revealing an ERBB2 CNG (112 by tumor tissue and 91 by ctDNA). The cancer subtypes with the most cases with ERBB2 CNG by NGS were breast (*n* = 67), non-small cell lung (*n* = 25), colorectal (*n* = 18), gastroesophageal (*n* = 17), pancreatic (*n* = 11), and uterine (*n* = 11). The PPV of ERBB2 CNG in determining HER2 positivity by standard IHC/FISH definitions was 88% for tissue NGS (*n* = 57) and 80% for ctDNA (*n* = 47). The PPV among breast cancer patients for tissue NGS was 97% (*n* = 35) and ctDNA was 93% (*n* = 39). However, for non-breast cancer cases, the PPV of ERBB2 amplification by tissue NGS dropped to 76% (*n* = 22) and by ctDNA to 44% (*n* = 7).

Conclusions: ERBB2 CNG by NGS is detected in numerous malignancies for which HER2 testing is not standard. Detection of ERBB2 CNG by tissue NGS and ctDNA has a high PPV for true HER2-positivity by standard IHC and/or FISH testing in breast cancer.

## INTRODUCTION

Trastuzumab’s approval in 1998 after it was shown to improve overall survival in HER2-postive metastatic breast cancer is one of the earliest successes in precision oncology and targeted therapies [1]. Since that time, advances in next-generation sequencing (NGS) technology have translated into the ability to efficiently study a broad array of molecular changes in patients with advanced cancer [2]. However, the clinical implications of gene alterations revealed by NGS in the context of a specific disease subtype or patient is often not known.

Overexpression of HER2, primarily as a result of amplification of *ERBB2*, identifies an aggressive breast cancer phenotype with a high risk for metastatic disease to visceral organs and the central nervous system. HER2-targeted therapies have dramatically changed the outcomes in patients with HER2-driven breast cancers, with the median overall survival for metastatic HER2-positive breast cancer reaching over 4 years and over one-third of patients surviving at 8 years [3]. HER2 overexpression and/or amplification is also seen in other cancers and has been shown to be predictive of trastuzumab efficacy for these diseases [4–6].

To date, all FDA-approved uses of HER2 targeted agents have been based on studies using HER2 immunohistochemistry (IHC) or fluorescence *in situ* hybridization (FISH) testing as a predictive assay to identify those who are likely to benefit from therapy [7]. Given the increasing access to and utilization of tumor tissue and ctDNA NGS in advanced malignancies, we aimed to better understand how *ERBB2* copy number gains (CNG) by commercial NGS platforms of tissue or blood predict for HER2-positivity by the ‘gold-standard’ of IHC and FISH assays. Additionally we aimed to describe malignancies with NGS HER2 CNG in tumor types for which HER2 testing by IHC or FISH is not routinely done.

## RESULTS

As of September 2019, the OncoSET database included 3337 patients with tissue NGS reports (FoundationOne or TempusX) and 1779 patients had ctDNA NGS testing (Guardant360). Of these patients, 177 patients were identified with CNG of *ERBB2* by tissue or blood-based NGS testing. Tissue NGS identified *ERBB2* CNG in 113 patients (3.3% of patients with tissue NGS reports, 82 on FoundationOne, 35 by TempusX, and 1 by both), and ctDNA NGS identified *ERBB2* CNG in 91 patients (5.2% of patients with NGS ctDNA testing). Twenty-seven patients had *ERBB2* amplification by both tissue NGS and ctDNA.

**Figure 1:**
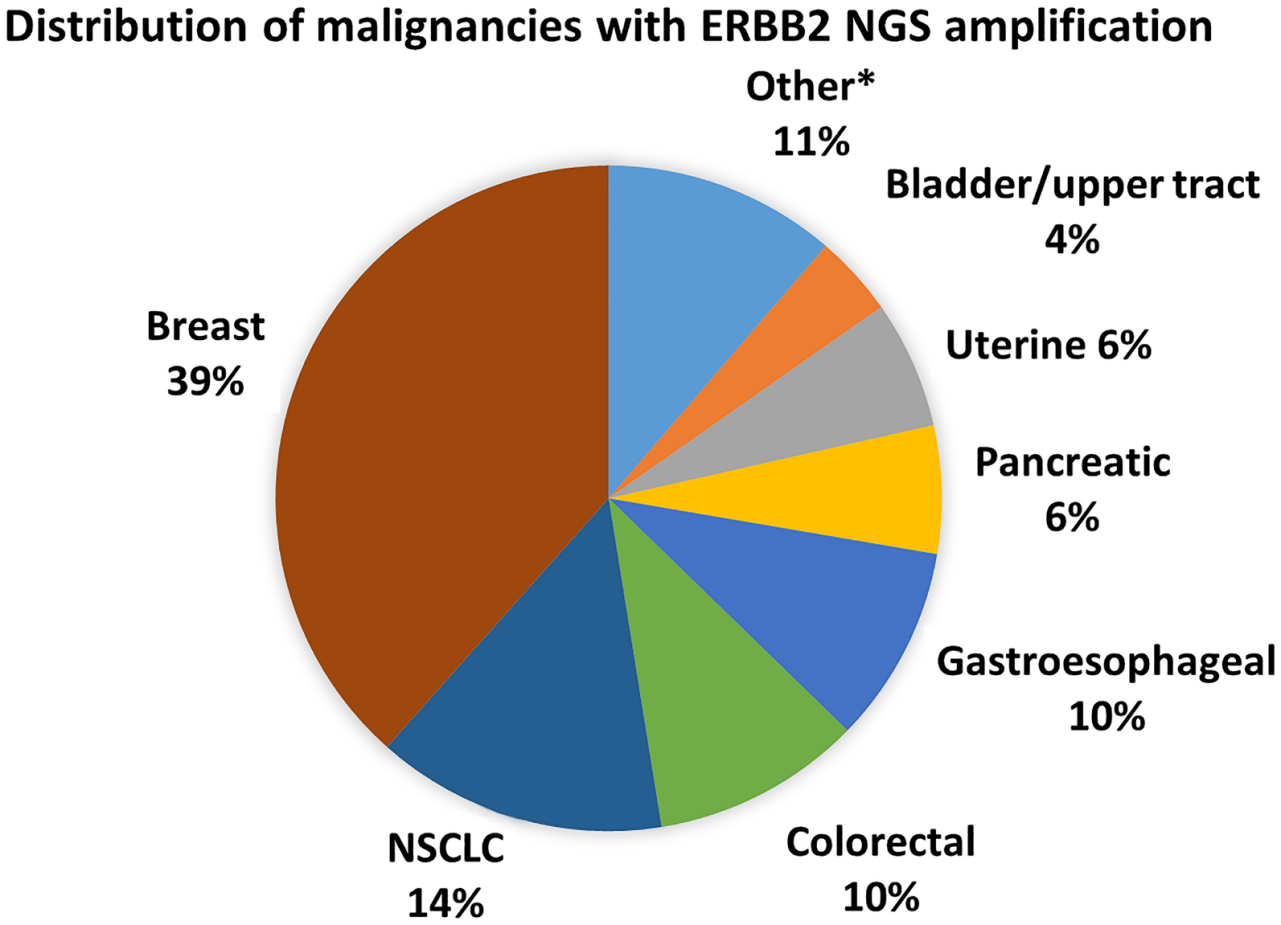
Cancer subtypes with ERBB2 CNG. ^*^Other includes ovarian/fallopian tube (4), biliary (3), SCLC (3), neuroendocrine (2), salivary (2), anal (1), cervical (1), melanoma (1), RCC (1), small bowel (1), unknown primary (1)

The median age at sample collection was 56 years (interquartile range 45–65 years), 69.5% were female, race was 73% White, 7% Asian, 12% Black, and 11% unknown, and 9% were Hispanic (Table 1). *ERBB2* CNG was seen in 18 subtypes of cancer (Figure 1), the most common malignancies of which were: breast (*n* = 68), non-small cell lung (NSCLC, *n* = 25), colorectal (*n* = 18), gastroesophageal (*n* = 17, 15 adenocarcinoma, 2 squamous), pancreatic (*n* = 11), uterine (*n* = 11), bladder/upper tract (*n* = 7), ovarian/fallopian tube (*n* = 4), biliary (*n* = 3), and small cell lung cancers (SCLC, *n* = 3) (Figure 1). *ERBB2* CNG was also seen in patients with anal cancer, carcinoma unknown primary, cervical cancer, melanoma, neuroendocrine cancer, renal cell carcinoma, and salivary gland cancer (*n* = 1 for each). The site of biopsy used for IHC and NGS testing, if tissue NGS testing was performed, was liver in 37%, lymph node in 18%, lung in 12%, bone in 7%, central nervous system in 5%, skin in 5%, with the remainder from other sites, most frequently breast.

**Table 1:** Clinical and pathologic characteristics of patients with ERBB2 CNG with HER2 tissue IHC and/or FISH testing and PPV of ERBB2 CNG for HER2-positivity

	Overall	Breast	GEA	Other
Age (Median, Interquartile Range)	54.9	51.5	64.8	65.0
Gender				
Female	94 (83.2%)	66 (98.5%)	6 (85.7%)	26 (66.7%)
Male	19 (16.8%)	1 (1.5%)	1 (14.2%)	13 (33.3%)
Race				
Asian	2 (1.8%)	1 (1.5%)	1 (14.3%)	0
Black or African American	14 (12.4%)	8 (11.9%)	0	6 (15.4%)
White	79 (69.9%)	46 (68.7%)	5 (71.4%)	28 (71.8%)
Unknown	17 (15.0%)	11 (16.4%)	1 (14.3%)	5 (12.8%)
Ethnicity				
Not Hispanic or Latino	97 (85.8%)	58 (86.6%)	7 (100%)	32 (82.1%)
Unknown	9 (8.0%)	4 (6.0%)	0	5 (12.8%)
Hispanic or Latino	7 (6.2%)	5 (7.5%)	0	2 (5.1%)
Biopsy Site				
Other	33 (29.2%)	15 (22.4%)	6 (85.71%)	12 (30.8%)
Liver	21 (18.58%)	9 (13.4%)	1 (14.29%)	11 (28.2%)
Lymph Node	16 (14.16%)	10 (14.9%)	0 (0.00%)	6 (15.4%)
Lung	14 (12.4%)	7 (10.5%)	0	7 (18.0%)
Bone	11 (9.7%)	10 (14.9%)	0	1 (2.6%)
Skin	8 (7.1%)	8 (11.9%)	0	0
CNS	8 (7.1%)	7 (10.5%)	0	1 (2.6%)
Breast	1 (0.9%)	1 (1.5%)	0	0
Colon	1 (0.9%)	0	0	1 (2.6%)
Total	113	67	7	39

Overall 60% of patients with *ERBB2* CNG by NGS testing had corresponding complete IHC/FISH, including 99% of patients with breast cancer, 40% of patients with GEA, and 35% of patients with other cancers (Table 2).

**Table 2:** Proportion of patients with *ERBB2* CNG with HER2 tissue IHC and/or FISH testing and PPV of *ERBB2* CNG for HER2-positivity

	NGS (tissue and/or ctDNA)		Tissue NGS		ctDNA	
	% complete IHC/FISH	PPV (%, 95% CI)	% complete IHC/FISH	PPV (%, 95% CI)	% complete IHC/FISH	PPV (%, 95% CI)
Overall	105/176 = 60%	86/105 = 82% (73%–89%)	65/112 = 58%	57/65 = 88% (77%–95%)	59/91 = 65%	47/59 = 80% (67%–89%)
Breast	66/67 = 99%	62/66 = 94% (85%–98%)	36/37 = 97%	35/36 = 97% (85%–100%)	43/44 = 98%	40/43 = 93% (81%–99%)
GEA	6/15 = 40%	5/6 = 83% (36%–100%)	6/14 = 43%	5/6 = 83% (36%–100%)	0/1 = 0	0 (0%–98%)
Other	33/94 = 35%	19/33 = 58% (39%–75%)	23/61 = 38%	17/23 = 74% (52%–90%)	16/46 = 35%	7/16 = 44% (20%–70%)

The PPV of *ERBB2* CNG for HER2-positivity was 82% (95% confidence interval 73%–89%) overall, 88% (77%–95%) for tissue NGS, and 80% (67%–89%) for ctDNA (Table 2). Among patients with breast cancer the PPV was 94% (85%–98%) overall, 97% (85%–100%) for tissue NGS and 93% (81%–99%) for ctDNA. Among patients with gastroesophageal adenocarcinomas, the rates of HER2 IHC and/or FISH testing was low (40%), but 5 of the 6 patients with tissue NGS amplification had HER2 IHC and/or FISH positivity. Among the non-breast, non-GEA patients, the PPV of tissue ERBB2 CNG was 74% (52%–90%) and of ctDNA was 47% (20%–70%).

Nineteen cases had discordant NGS and IHC/FISH results (Table 3). All had IHC testing which showed no HER2 expression in 11, and HER2 1+ in 8. All of the patients showing discordance with *ERBB2* amplification by tissue NGS but lack of HER2 overexpression by IHC (*n* = 8) had testing done from the same biopsy. For the 11 cases with discordance by ctDNA analysis and IHC, the median time between the biopsy used for IHC testing and ctDNA analysis was 0 months, with a range of 0–25 months).

**Table 3:** Cases with discordant results in HER2 by NGS and IHC/FISH

Disease subtype	IHC score	FISH ratio	HER2 copy number	ASCO/CAP classification	Tissue NGS ERBB2 CNG, laboratory performing NGS	ctDNA ERBB2 amp	Months between tissue for HER2 IHC/FISH & NGS
Breast	0	−	−	Negative	−	+	1
Breast	0	−	−	Negative	−	++	25
Breast	0	−	−	Negative	−	+++	0
Breast	1+	1.35	4.6	Group 4, negative with comment	CNG, Tempus	−	0
NSCLC, adenocarcinoma	0	−	−	No standard	CNG, Foundation	+	0
NSCLC, squamous	1+	−	−	No standard	−	++	0
NSCLC, neuroendocrine	0	−	−	No standard	CNG, Foundation	−	0
NSCLC, adenocarcinoma	1+	−	−	No standard	−	+	6
NSCLC	1+	−	−	No standard	−	+	0
NSCLC, squamous	0	−	−	No standard	−	++	12
Colorectal	0	−	−	No standard	CNG, Tempus	−	0
Colorectal	0	−	−	No standard	CNG, Foundation	−	0
Pancreatic	0	−	−	No standard	−	++	10
SCLC	0	−	−	No standard	−	++	0
Esophageal, adenocarcinoma	1+	−	−	Negative	CNG, Foundation	−	0
Esophageal, squamous	1+	−	−	Negative	−	+	0
RCC	1+	−	−	No standard	CNG, Foundation	−	0
Urothelial	1+	−	−	No standard	CNG, Foundation	−	0
Ovarian	0	−	−	No standard	−	++	0

Discordant cases included 4 breast cancer cases, 3 of which lacked HER2 overexpression but had CNG of varying degrees by ctDNA. The remaining discordant breast cancer case had tissue NGS amplification and HER2 copy number >4, but given the lack of overexpression and HER2/CEP17 ratio <2.0, was negative per guidelines for patients falling into Group 4 with the comment “it is uncertain whether patients […] benefit from HER2 targeted therapy in the absence of protein overexpression”. One discordant case also harbored a missense single nucleotide polymorphism in *ERBB2* of unknown significance.

Eight cases had equivocal HER2 IHC/FISH results. This included 7 non-breast, non-GEA cases with an equivocal IHC score (2+) but no FISH testing. One case of breast cancer had a HER2 copies of 35 but HER2/CEP17 ratio of only 1.04, meeting Group 3 criteria. However, subsequent IHC testing was not completed, so the case remained equivocal. Of note, about half (53%) of all patients with NGS *ERBB2* amplification had IHC testing, of which 88% had at least some HER2 expression (IHC score 1+, 2+, or 3+). The PPV for any level of HER2 IHC expression was 95% for breast, 100% for GEA, and 73% for non-breast non-GEA.

## DISCUSSION

The utilization of NGS testing in advanced malignancies continues to increase dramatically as options for targeted therapies have expanded and accessibility, affordability, and efficiency of NGS testing have improved [8, 9]. As a result, *ERBB2* CNG may be discovered by NGS testing but its correlation with standard HER2 testing by IHC for overexpression or FISH for amplification, which have been validated in breast and GEA cancers to predict benefit from HER2-therapy, is less well understood.

Our study demonstrated *ERBB2* CNG found on NGS testing across over 10 different tumor types. Over half of the cases of ERBB2 CNG were found in tumors for which HER2 testing is not routine and there is no standard HER2 IHC/FISH cut offs. This is consistent with data shown in large metastatic cancer NGS databases [10–12]. This is noteworthy because of rapid growth in therapeutics targeting HER2 and studies suggesting the impact of HER2 therapies can be seen in multiple cancer subtypes with HER2 overexpression or amplifications [13–17].

Our retrospective study showed *ERBB2* CNG in breast cancer cases has a high PPV for HER2-positivity by IHC/FISH by ASCO/CAP 2018 guidelines (PPV for tissue NGS 97%, ctDNA 93%). This finding is consistent with reported data regarding the high concordance of *ERBB2* CNG by tissue NGS with the MSK-IMPACT assay and HER2 IHC/FISH testing [18]. Additionally, the rates of false positive *ERBB2* amplification by NGS for breast cancer (6%) is similar to inter-laboratory differences seen with IHC/FISH testing for breast cancer [19]. The high PPV of *ERBB2* CNG for HER2-positivity in breast cancer is particularly relevant as tissue biopsies can be a challenge for patients with bone predominant disease. Not only are these tissue biopsies often challenging to obtain, but the processing required for these biopsies (i.e. decalcification) interferes with both the IHC and FISH assays, and these assays are often not validated for use on decalcified specimens, with a higher risk for a false negative on decalcified specimens. Additionally, HER2 heterogeneity and tumor evolution may mean the HER2 IHC/FISH results from an initial biopsy or surgical resection specimen may not accurately reflect a patient’s current HER2-status, especially given the relatively long disease course of many patients with advanced breast cancer. One recently published breast cancer cohort also suggested high concordance between tissue IHC/FISH and *ERBB2* CNG by ctDNA [20]. Together, these findings suggest that ctDNA *ERBB2* CNG is likely to be an accurate marker of HER2-positivity as determined by traditional assays. If ctDNA reveals *ERBB2* CNG discordant from a prior HER2-negative tissue IHC/FISH, it may prompt consideration of a new biopsy and/or use of HER2 therapy as the discordance may be a result of HER2 heterogeneity or tumor evolution rather than assay variability.

Our study was limited by a low proportion of patients with GEA having IHC/FISH testing. Nevertheless, in GEA with testing available, *ERBB2* CNG by tissue NGS had a relatively high PPV (83%) for HER2-positivity by ASCO/CAP guidelines. This is consistent with reported findings of a high concordance between tissue NGS *ERBB2* CNG and tissue IHC/FISH in GEA [18, 21–24]. Unfortunately, we did not identify patients with GEA ctDNA *ERBB2* CNG and IHC and/or FISH.

For non-breast, non-GEA cancers, only 35% had IHC and even fewer had FISH testing. In the group that had IHC and/or FISH, the discordance was higher, with a PPV of only 58% overall and 74% for tissue NGS and 44% for ctDNA. However, this discordance is primarily in *ERBB2* CNG by NGS and HER2 overexpression by IHC (IHC 3+) because only 7 of 33 non-breast non-GEA cases included in the PPV analysis had FISH testing. This discordance in *ERBB2* amplification by sequencing and HER2 protein expression has been shown in several tumors types [13, 14]. In colon cancer, NGS testing has been shown to have good concordance with tissue IHC/FISH testing although criteria for HER2-positivity in colon cancer have yet to be standardized [25, 26]. If similar findings are confirmed across tumor type, discovering tissue *ERBB2* CNG by NGS can facilitate clinical trial enrollment and utilization of HER2-thearpy in diseases for which HER2 testing by IHC/FISH is not standard. Of note, in a study of 57 patients with metastatic colorectal cancer and *ERBB2* amplification, despite an overall objective response rate of 32%, none of the 8 patients with amplification without HER2 overexpression responded to trastuzumab and pertuzumab.

One potential reasons for the lower PPV observed in non-breast cases include a chance finding due to small numbers. Another possible explanation is there is a lower prevalence of biologically important amplifications in HER2 in other malignancies, thus a lower pre-test probability and a higher risk of a false positive result.

The limited number of patients with non-breast malignancies who had ctDNA *ERBB2* CNG and tissue IHC/FISH prevents adequate evaluation of its PPV in these cohorts. Nevertheless, if the findings seen in breast cancer patients are replicated in other malignancies it could provide significant clinical value. Challenges of HER2 testing include disease heterogeneity and adequate tissue sampling, both areas where ctDNA may offer advantages in a wide variety of tumor types [27]. Thus, future studies of *ERBB2 CNG* by ctDNA and HER2 IHC/FISH tissue correlation in both breast and non-breast cancer are needed. Such a study may also investigate whether co-alterations or other molecular signatures present along with *ERBB2* CNG that could predict the likelihood that the ERBB2 CNG is reflective of a true biologically-relevant amplification likely to benefit from HER2 therapy.

One limitation of the study is our inability to determine the prevalence of *ERBB2* alterations by tumor type as some tumors where NGS testing has been commonplace for longer, such as NSCLC, are likely to be over-represented in our database [8]. Another important limitation of our study is the limited number of non-breast patients who had IHC/FISH testing, making it challenging to determine an accurate PPV in these settings. Additionally, as we queried only those with *ERBB2* CNG, we are able to determine the PPV but not the sensitivity or negative predictive value. Disadvantages of the use of NGS testing for *ERBB2* evaluation are the presence of false positives, its reliance on the availability of tissue (more tissue sections required than IHC) or shedding of ctDNA, added cost, lack of insurance coverage, and added turnaround time.

A primary challenge in optimizing HER2 targeted therapy is accurately identifying patients who will benefit from treatment. HER2-positivity by IHC/ISH ASCO/CAP guidelines is based on their predictive value for trastuzumab efficacy in breast and GEA cancers, but likely does not capture the breadth of metastatic cancer patients who may benefit from the remarkable efficacy our expanding arsenal of HER2 agents that possess different activity profiles to trastuzumab. Additionally, IHC/FISH testing of a single, often archival biopsy specimen may not adequately capture tumor heterogeneity or evolution of HER2 status and may not provide the optimal threshold for HER2-positivity in the setting of newer therapy. As we study HER2 activity in novel settings with novel drugs (including trastuzumab-deruxtecan and other antibody-drug conjugates with activity in HER2-low settings), keeping a broad view of methods of evaluating HER2 (tissue IHC, tissue FISH, tissue NGS, ctDNA, expression on circulating tumor cells), their correlation, and their ability to predict benefit is important [28, 29]. In the setting of anti-HER2 therapy with activity in HER2-low disease, the PPV of NGS *ERBB2* amplification for any level of HER2 IHC expression reached 88%.

As we move into our third decade of developing HER2 therapy at a time where tumor agnostic treatment approaches and NGS testing in metastatic cancer is common, utilizing *ERBB2* CNG by NGS to identify patients for clinical trials evaluating novel HER2 therapies in new disease settings may be a more efficient screening strategy, and has been employed in the MyPathway (NCT 02091141) and NCI-MATCH (NCT 02465060) trials successfully. This is especially relevant in tumors types that have a lower prevalence of *ERBB2* amplification. Future efforts should look to validate the high PPV of *ERBB2* CNG by NGS for HER2-positive IHC/FISH in breast cancer and other tumor types and subsequently whether *ERBB2* CNG is predictive of benefit from HER2-targeted therapy.

## MATERIALS AND METHODS

The study was approved by the institutional review board and the requirement for informed consent was waived for this retrospective analysis. We queried our OncoSET database to identify patients within a single hospital system (Northwestern Medicine) who had NGS performed by 3 commercial laboratory assays (FoundationOne and TempusX for tissue NGS, or Guardant360 for ctDNA) that revealed a CNG or amplification in *ERBB2* on the issued report between 2015 and 2019.

Clinical and pathologic information was subsequently gathered by review of the electronic medical record. The data obtained included patient demographic information and disease information including type of malignancy, histology, stage, detailed HER2 IHC and FISH testing when available, and other genomic alterations detected. Time course of disease was collected including date of diagnosis of advanced malignancy and date of sample collection for NGS. When possible, tissue was retrieved for HER2 IHC testing among patients with *ERBB2* NGS amplification but without IHC/FISH results. If IHC/FISH data were available for patients with breast cancer or gastroesophageal adenocarcinoma (GEA), the patient was classified as HER2-positive by ASCO/CAP guidelines [7, 30]. For all non-breast, non-GEA patients HER2-positivity was defined as IHC 3+, FISH ratio of >2.0, and/or HER2 copy number >6, similar to breast cancer and GEA criteria. Cases in which IHC score was 2+ and FISH was not available or FISH was equivocal and IHC was not available were considered equivocal.

Descriptive statistics were calculated for patient and disease characteristics of those identified as harboring an *ERBB2* CNG. The positive predictive value (PPV) of *ERBB2* CNG by NGS in predicting HER2-positivity by IHC/FISH was calculated by the overall population, type of sample (tissue NGS vs. ctDNA NGS), and by disease type (breast, GEA, other). If IHC/FISH results were equivocal because of incomplete testing, the cases were excluded from calculations of PPV. Binomial proportion was used to determine the 95% confidence interval.

## Abbreviations

CI: confidence interval
CNG: copy number gain
ctDNA: circulating tumor DNA
FISH: fluorescent *in situ* hybridization
GEA: Gastroesophageal adenocarcinoma
IHC: Immunohistochemistry
NGS: Next-generation sequencing
PPV: positive predictive value
